# Indel sensitive and comprehensive variant/mutation detection from RNA sequencing data for precision medicine

**DOI:** 10.1186/s12920-018-0391-5

**Published:** 2018-09-14

**Authors:** Naresh Prodduturi, Aditya Bhagwate, Jean-Pierre A. Kocher, Zhifu Sun

**Affiliations:** Division of Biomedical Statistics and Informatics, Department of Health Sciences Research, Mayo Clinic, 200 First St SW, Rochester, MN 55905 USA

**Keywords:** RNA sequencing, Somatic mutations, Insertion/deletion, Fusion transcript, Gene expression, Targeted therapy, Precision medicine

## Abstract

**Background:**

RNA-seq is the most commonly used sequencing application. Not only does it measure gene expression but it is also an excellent media to detect important structural variants such as single nucleotide variants (SNVs), insertion/deletion (Indels) or fusion transcripts. However, detection of these variants is challenging and complex from RNA-seq. Here we describe a sensitive and accurate analytical pipeline which detects various mutations at once for translational precision medicine.

**Methods:**

The pipeline incorporates most sensitive aligners for Indels in RNA-Seq, the best practice for data preprocessing and variant calling, and STAR-fusion is for chimeric transcripts. Variants/mutations are annotated, and key genes can be extracted for further investigation and clinical actions. Three datasets were used to evaluate the performance of the pipeline for SNVs, indels and fusion transcripts.

**Results:**

For the well-defined variants from NA12878 by GIAB project, about 95% and 80% of sensitivities were obtained for SNVs and indels, respectively, in matching RNA-seq. Comparison with other variant specific tools showed good performance of the pipeline. For the lung cancer dataset with 41 known and oncogenic mutations, 39 were detected by the pipeline with STAR aligner and all by the GSNAP aligner. An actionable EML4 and ALK fusion was also detected in one of the tumors, which also demonstrated outlier ALK expression. For 9 fusions spiked-into RNA-seq libraries with different concentrations, the pipeline was able to detect all in unfiltered results although some at very low concentrations may be missed when filtering was applied.

**Conclusions:**

The new RNA-seq workflow is an accurate and comprehensive mutation profiler from RNA-seq. Key or actionable mutations are reliably detected from RNA-seq, which makes it a practical alternative source for personalized medicine.

**Electronic supplementary material:**

The online version of this article (10.1186/s12920-018-0391-5) contains supplementary material, which is available to authorized users.

## Background

Somatic mutations are a hallmark of a tumor and inherited mutations cause certain genetic disorders. Characterization of these mutations and exploration of their clinical relevance constitute a critical part of personalized medicine. Mutations present in multiple forms and common ones include single nucleotide variants (SNVs), short insertions/deletions (indels), or fusion transcripts. SNVs or indels are primarily detected from DNA sequencing such as whole-genome, exome-sequencing, targeted sequence or amplicon. However, RNA-seq is the most popular sequencing application as it contains much richer genomic information. Not only does it measure gene expression but it also can detect important structural variants such as SNVs, indels or fusion transcripts, some of which are known actionable mutations for tumor treatment. A good example of this is EGFR single base mutation (L858R) in exon 21 and in-frame deletions (ranging from 12 to 18 bases) in exon 19, both can be targeted by EGFR tyrosine kinase inhibitors, such as gefitinib and erlotinib with clear clinical benefits to patients [1]. Although fusion transcript detection from RNA-seq is commonly used [2–4], use of RNA-seq for SNV or Indel mutation detection in clinical settings is still rare, which is contributed by several reasons. Detection of structural variants from RNA-seq is much more challenging. RNA transcripts are spliced molecules from different parts of genome and exon-exon junction aware alignment is needed. This alignment causes difficulty for variant calling tools, which are mostly developed for DNA-sequencing. As the primary goal of RNA-seq is gene expression profiling, commonly used RNA-seq mapping programs often conduct ungapped mapping and sequence reads with insertion or deletion are un-mappable and these variants would be ignored [5]. Even for the same alignment, variant calling tools perform differently, particularly for Indel detection [5]. Another concern for RNA-seq based mutation detection is differential gene expression, which leads to variable coverage between genes and affects variant detection for genes expressed at low level. This is highly relevant and important for mutation discovery. Meanwhile, data also show that key or driver mutations often occur in expressed genes and tend to be conserved and easily detectable in RNA-seq [6], which makes RNA-seq based mutation detection a potential cost effective alternative if it can be used for multiple information profiling simultaneously.

Many RNA-seq workflows have been developed [7–9], but they mostly perform a particular function in research settings. MAP-RSeq [8] is a comprehensive analytical pipeline with gene expression quantification, fusion transcript and SNV detection, but it cannot detect indels. PRADA [7] focuses fusion detection and annotation. A recent tool Opossum [10] conducts comprehensive RNA-seq alignment pre-processing before variant calling by either Platypus [11] or GATK Haplotype Caller [12] but only SNVs are evaluated. As continuation of our previous work in detecting Indels from RNA-seq, we have developed an integrated RNA-seq pipeline “PanMutsRx” with goal of reporting common and clinical important mutations (SNVs, indels, fusion transcript) at once. PanMutsRx implements RNA-seq alignment programs that conduct gapped and junction aware mapping, performs rigourous pre-processing steps unique to RNA-seq before variant calling, incorporates selected best performing single sample variant and paired somatic mutation callers, and optionally reports mutations for a list of genes in interest. Using a sample from Genome in a Bottle Consortium where variants are well defined from multi-platform DNA-sequencing, we demonstrated its good performance in SNV and Indel detection. We also tested a set of clinical samples with known mutations and fusion transcripts and showed that important mutations were almost all detectable, which makes it a potential application for clinical applications.

## Methods

### Pipeline implementation

**Fig. 1:**
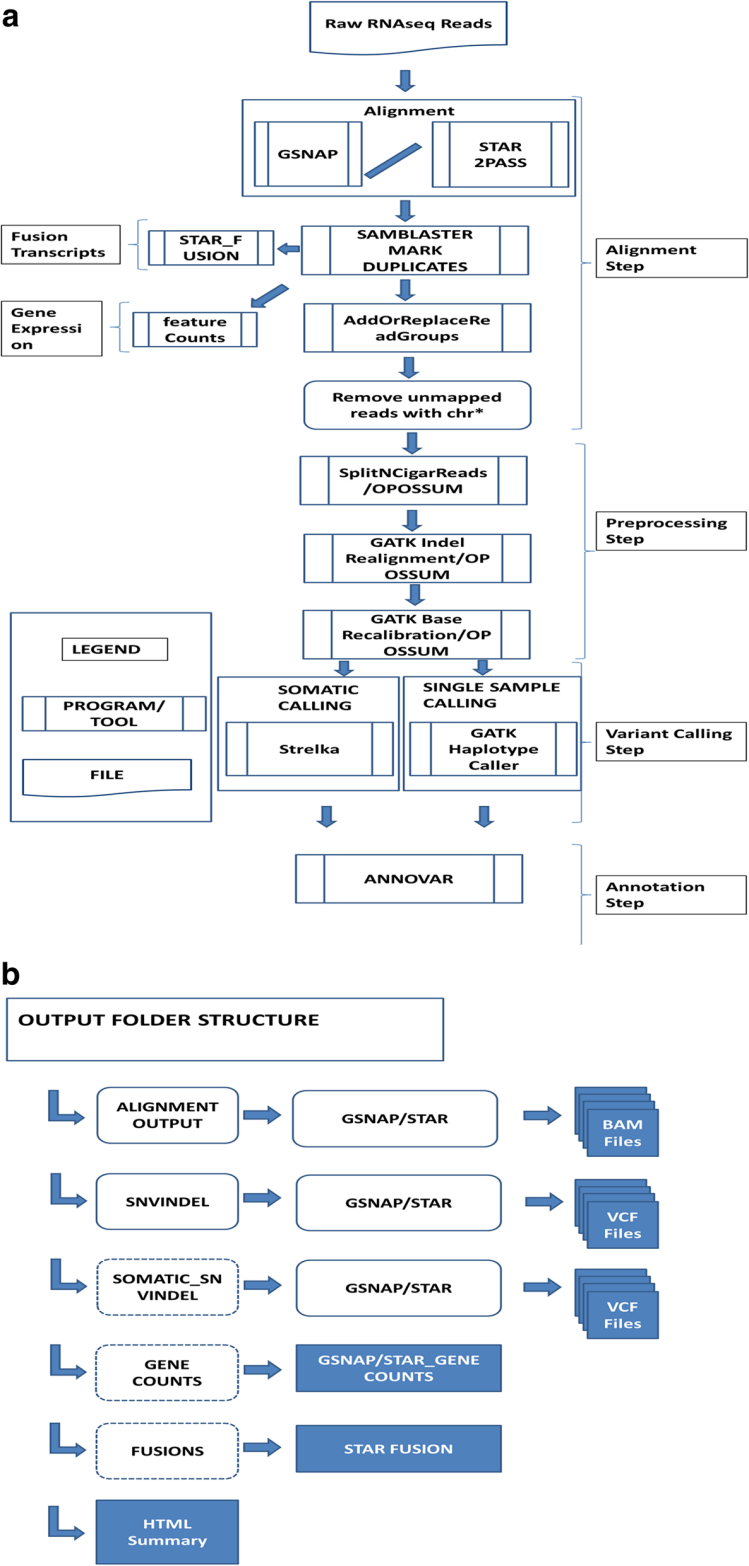
PanMutsRx workflow. **a** workflow diagram. **b** output structure

PanMutsRx is implemented modularly using Python 3 and shell scripts for various operations such as input/output file operations, log file management, submitting jobs to cluster (optional), tool execution and integration. The main operation of the workflow is summarized in Fig. 1a.

A. **Sequence read alignment**: This pipeline includes two aligners i.e. GSNAP [13] and STAR [14], with STAR as default (or both can be run at the same time). STAR is an ultrafast RNA-Seq junction aware aligner which uses sequential maximum mappable seed search, seed clustering and stitching. A two-step alignment is implemented to increase the accuracy; in the first step splice junctions are detected and are used to guide in the second alignment. STAR is not only superfast but also very sensitive for Indel detection as demonstrated in our previous work [5]. GSNAP is another junction aware and fast aligner which is tolerant to complex genomic events like variants and indels and was shown more sensitive for longer indels when sequence reads are short [5]. Read group information is added and duplicate reads are tagged with SAMBLASTER tool [15].
B. **Aligned read preprocessing for SNV/Indel detection**: RNA-Seq variant calling is much complex than DNA-seq and the gapped alignment also causes incompatibility with existing variant callers. This module prepares the aligned bam file for next variant calling step. SplitNCigar, Indel Realignment and Base Recalibration are done by GATK tool kit [12]. In the SplitNCigar step, reads are split into exon segments and sequences which overhang in the intronic region are hard clipped.
C. **Variant/somatic mutation Calling**: Our previous work showed GATK [12] haplotype caller performed superiorly for single sample mode SNV and indel detection, and Strelka [16] was better for paired tumor/normal somatic mutation calling in RNA-seq [5]. They are implemented as SNV and somatic caller, respectively.
D. **Variant annotation**: Functional annotation is a key step for identified variants to understand the potential clinical impacts. Annovar [17] is a lightweight, simple to use, and efficient tool to annotate variants. Annovar is integrated as part of workflow for variant interpretation.
E. **Fusion Transcripts**: Fusion transcripts are characteristic of tumors and highly relevant to targeted therapy [18, 19]. It is important to detect potentially targetable fusions for guided therapy. To provide seamless integration, STAR-Fusion is incorporated for this function.
F. **Gene Expression**: The gene level quantification is done using featureCounts software [20]. The gene expression data can be used for outliner gene expression detection or differential expression analysis where both raw digital read count and normalized RPKM expression are generated.
G. **Summary Report and output structure:** The output file structure is illustrated in Fig. 1b. The read alignment files are provided in the BAM file format and are indexed to view in the IGV, Single sample variant and somatic variant calls are in the VCF format, Fusion transcripts are provided in tab separated files and gene expression is represented as raw counts and RPKM values in tab separated files.

The workflow has flexibility to execute individual modules separately and appropriate log files are generated for troubleshooting. Additional options are provided to run the workflow in the open grid engine parallel cluster environment, but depending on the other grid engine types changes may need to be made. Parameters used for all steps are provided in Additional file 1: Table S1.

### Test data and pipeline evaluation

To evaluate the performance of PanMutsRx in SNV/Indel and fusion detection, we used 3 datasets.

#### Hapmap NA12878 RNA-Seq and DNA-Seq dataset

Genome in a Bottle (GIAB) consortium released a benchmark SNP and indel dataset for sample NA12878 by integrating multiple DNA sequence data sets including whole genome sequencing [21]. For the same sample, RNA-seq was performed through ENCODE project. We downloaded the raw RNA-seq data (https://www.encodeproject.org/; ENCFF377UIC with 147 million pair-end reads at 100 bp read length) and analyzed through our pipeline for SNVs and Indels and compared with the benchmark DNA variants. As variants from RNA-seq are only possible from coding regions and only expressed genes can be assessed, the comparison was limited to the genomic positions with at least 10X coverage in the RNA-seq where variants are reported in the reference dataset from GIAB. The sensitivity was calculated as the percent of correct calls in RNA-seq at these positions in comparison with variants in DNAs (SNVs or indels separately). For specificity, we extracted all positions in RNA-seq with at least 10X coverage but there are no variants in DNA as defined in GIAB benchmark set (true negatives or TN). Any variants in these positions reported from RNA-seq were considered as false positives (FP) and the specificity was obtained by the formula: TN/(TN + FP).

We also run sample ENCFF377UIC by other public tools and compared the relative performances for the SNV and Indel detection. Opossum is a RNA-seq preprocessing tool before variant calling by either Platypus or GATK haplotype caller and demonstrates a good performance in SNV detection [10]. In addition to the GATK best practices for RNA-seq variant calling [22], which PanMutRx follows, Opossum merges overlapping reads and modifies the base qualities at the ends of these reads before splitting them. Opossum can use Tophat or STAR alignment but we used the latter as the former does not allow Indel detection. RVBoost along with MAP-RSeq [8] is a RNA variant prioritization method with demonstrated better performance [23]. It uses several attributes unique for RNA-seq and a boosting method to train a model with reliable variants and then prioritizes the RNA SNV variants based on the trained model.

#### Lung cancer adenocarcinoma RNA-seq datasets with known oncogenic or targetable mutations

Lung cancer is one of tumors harboring a high number of mutations [24] and some of the mutations are sensitive to targeted therapy such as EGFR single nucleotide mutation at exon 21 (L858R) and intermediate indels (12 to 18 bases) at exon 19 [25] targeted by tyrosine kinase inhibitors [1] and EML4-ALK fusion targeted by kinase inhibitor Crizotinib [26]. The diverse cancer mutations and high yield targeted therapy provide an excellent use case to demonstrate the usability of PanMutsRx pipeline. To this end, we downloaded a lung adenocarcinoma dataset from SRA (ERP001058) consisting of 77 tumor and normal pairs with RNA-seq performed [27] where all of the aforementioned mutations are known to be present. The RNA-seq was sequenced at pair ends of 101 cycles and was analyzed in the paired mode for somatic mutations (comparing each tumor with its paired normal sample from each patient). The somatic mutations were compared with the known mutations.

#### Synthetic spike-in cancer gene fusions of mRNA-seq data

This publicly available dataset is created for the community to evaluate fusion detection algorithm where 9 well known oncogenic fusion transcripts were spiked into RNA-seq libraries at wide range of molarities [28]. We downloaded and evaluated 6 samples at the concentration of − 3.47, − 4.17, − 5.87, − 6.17, − 6.87, and − 8.57 through our pipeline. To reduce high false positives, we applied the filters that require combined normalized split and spanning fragment reads greater than 0.1 FFPM (J_FFPM + S_FFPM > 0.1, i.e., fusion fragments per million total reads) and the split reads are supported with at least 25 bases at both sides of a putative breakpoint. (“LargeAnchorSupport”==“YES_LDAS”).

## Results

### Comparison of SNVs and Indels detected from RNA-seq with golden standard DNA-seq of Hapmap NA12878

**Fig. 2:**
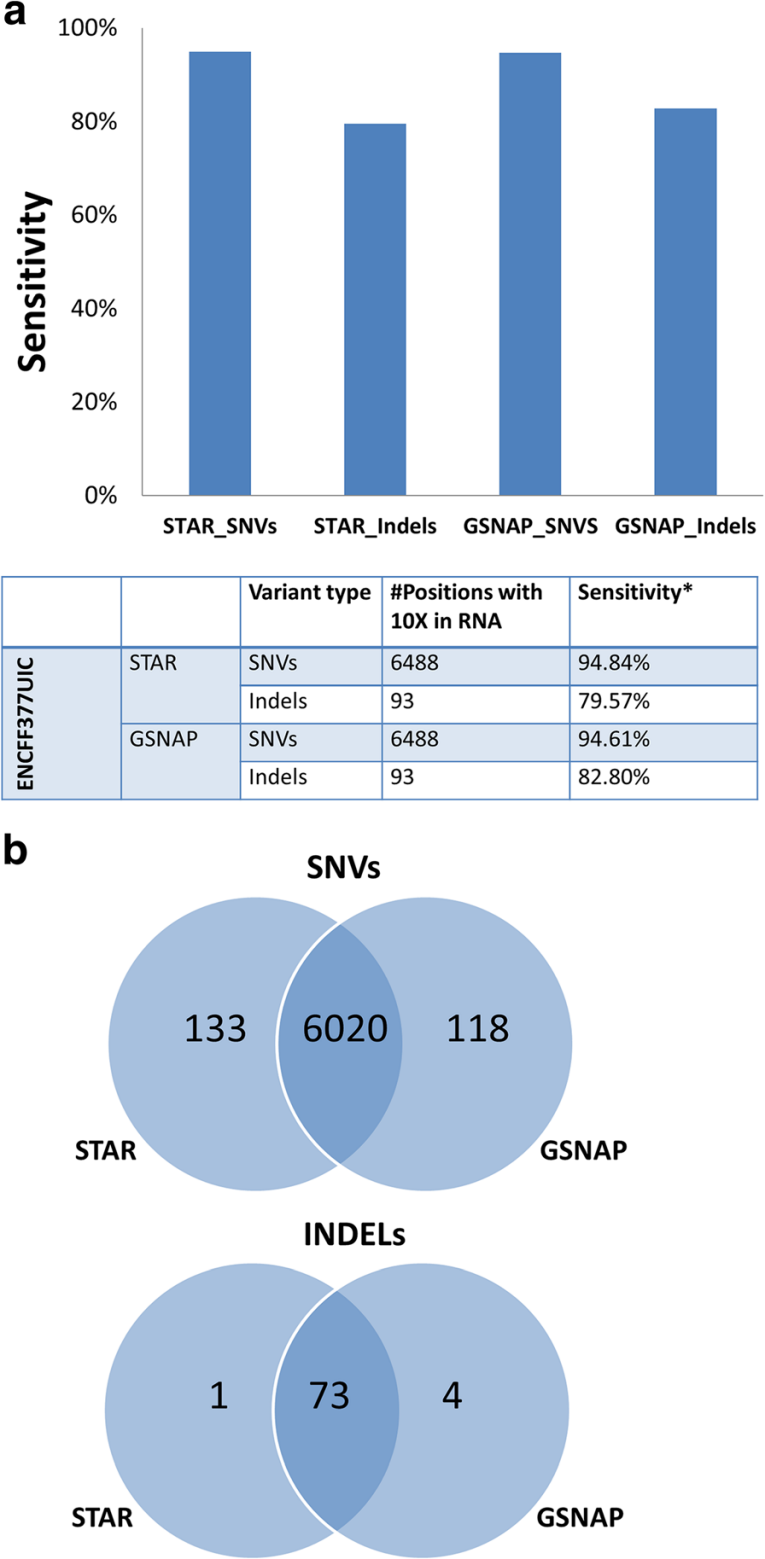
SNV and Indel detection by STAR and GSNAP alignment from PanMutsRx. **a** SNV and Indel detection sensitivities by STAR and GSNAP alignment. The sensitivity was based on the golden truth set in DNA from GIAB with > = 10X coverage in RNA-seq. **b** Overlap SNV and Indel calls between STAR and GSNAP alignment algorithm. STAR detected more true SNVs and GSNAP detected more true Indels

It took about 20 h for PanMutsRx to complete all the processing and analyses for the sample with 150 million reads (Additional file 1: Table S2). For GIAB reference variants detected from DNA, 6488 and 93 positions are covered with at least 10 reads for SNVs and Indels, respectively, in the RNA-seq library of ENCFF377UIC by the STAR alignment. PanMutsRx correctly detected 94.84% of SNVs and 79.57% of Indels (Fig. 2a). The similar results were observed from the alignment by GSNAP. A slightly higher concordance for SNPs was observed with STAR alignment whereas GSNAP was slightly more sensitive to indels, as overserved previously [5]. High concordance was also obtained for the variant calls between STAR and GSNAP alignments (Fig. 2b). About 98% SNVs and Indels called by either were common and consistent between the two aligners. As STAR is much faster than GSNAP and its alignment can be used for fusion transcript detection, our comparison hereafter used STAR alignment only.

#### Performance comparison with other RNA-seq variant calling tools

**Fig. 3:**
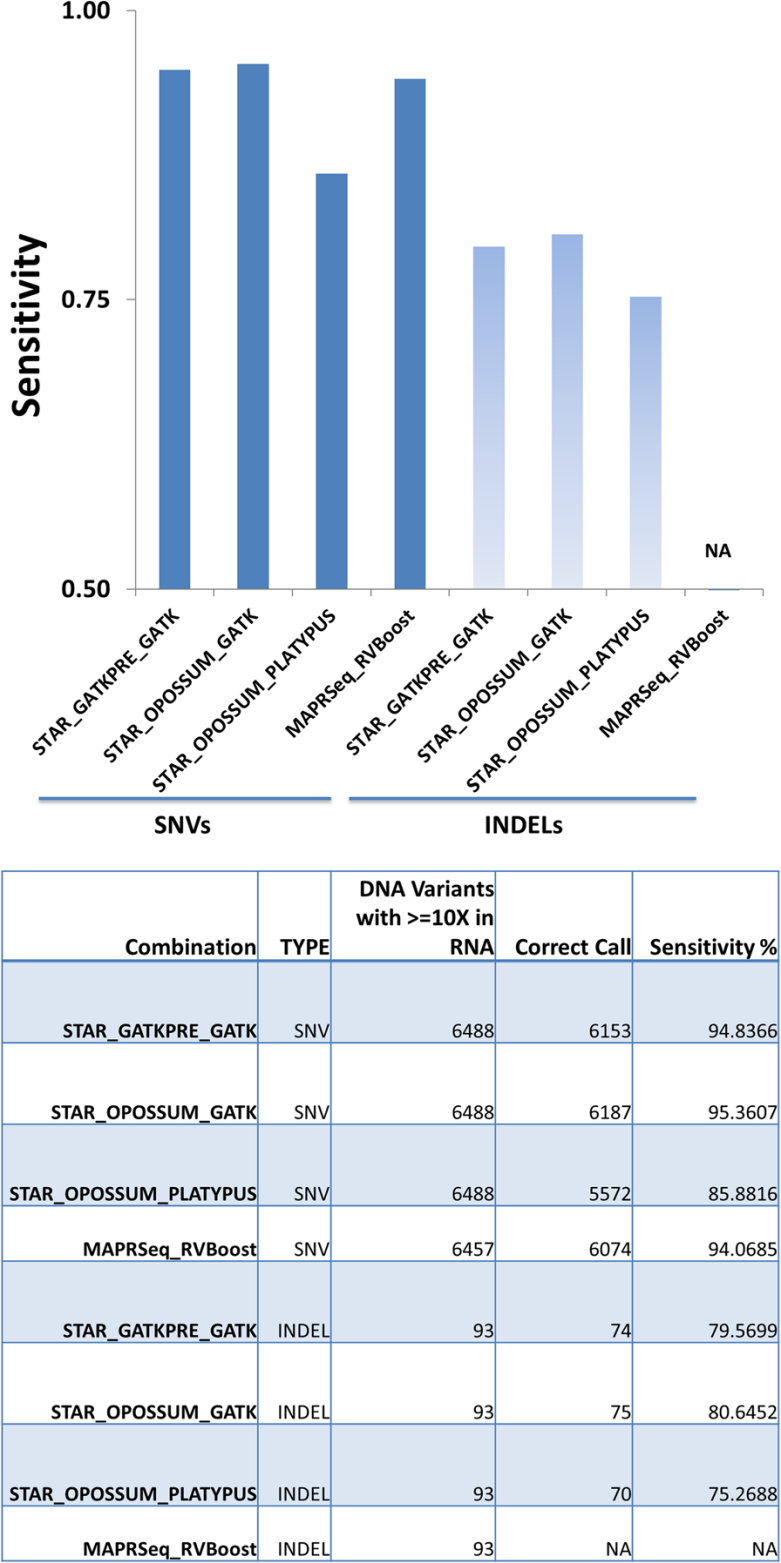
SNV and Indel sensitivity comparisons with other RNA-seq variant calling programs. STAR alignment was used for all except MAPRSeq_RVBoost, which used Tophat alignment. STAR alignment was pre-processed by PanMutsRx or Opossum and then variants were called by either GATK haplotype caller or Platypus. MAPRSeq_RVBoost variants were called by GATK unified genotyper. Default settings were used for variant calling and filtering. Sensitivity was calculated by dividing the number of correct calls in RNA by the total number DNA variants with 10X coverage in RNA-seq

**Fig. 4:**
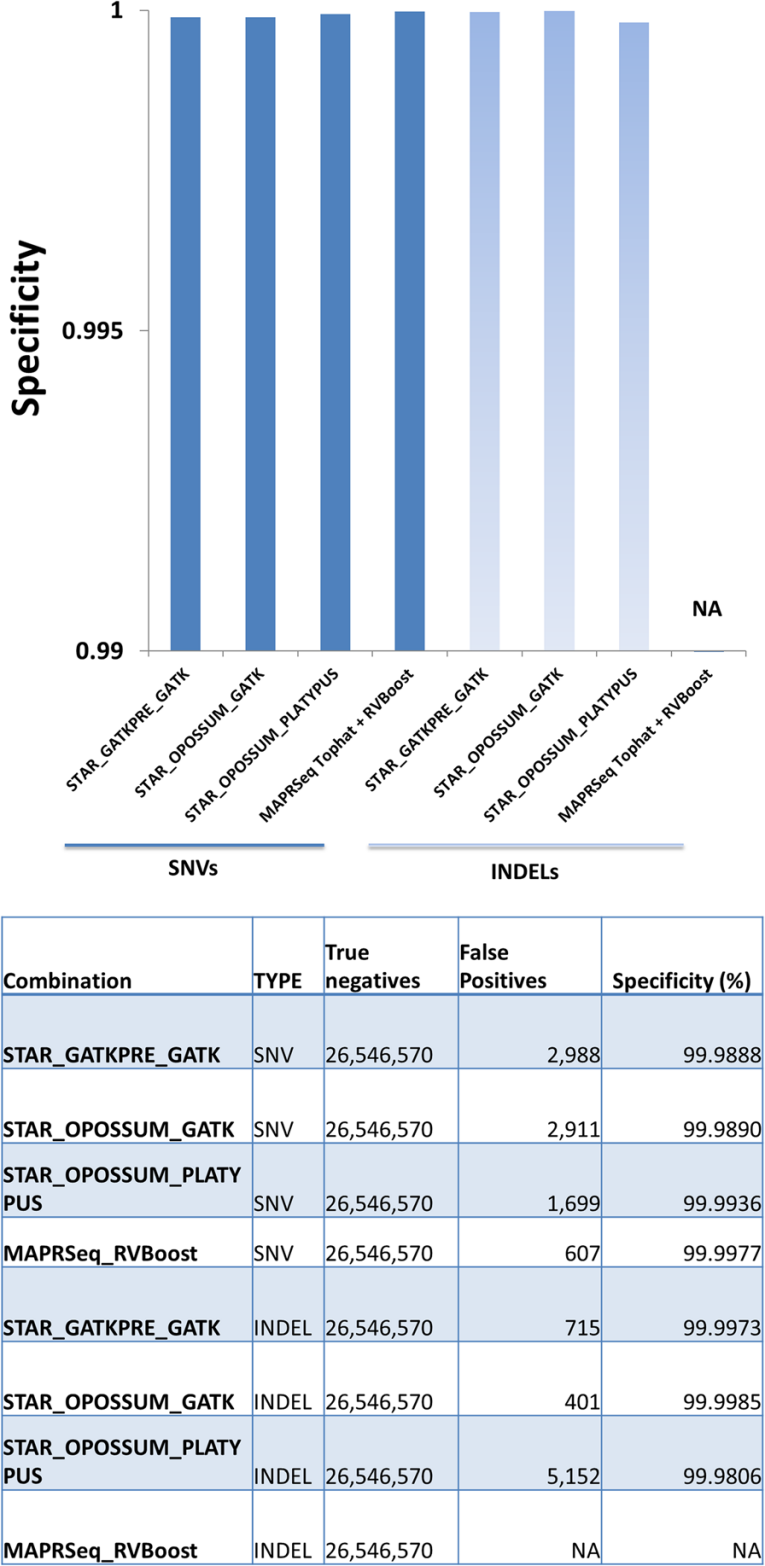
SNV and Indel specificity comparisons with other RNA-seq variant calling programs. STAR alignment was used for all except MAPRSeq_RVBoost, which used Tophat alignment. STAR alignment was pre-processed by PanMutsRx or Opossum and then variants were called by either GATK haplotype caller or Platypus. MAPRSeq_RVBoost variants were called by GATK unified genotyper. Default settings were used for variant calling and filtering. Specificity was calculated by TN/(TN + FP). TNs are the genomic positions with > = 10X coverage in RNA-seq calls but no variants present in DNA

We compared the variant results of PanMutsRx, which uses STAR alignment with GATK best practices preprocessing and GATK haplotype caller (STAR_GATKPRE_GATK), with STAR alignment by Opossum preprocessing and GATK haplotype caller (STAR_OPOSSUM_GATK), STAR alignment by Opossum preprocessing and PLATYPUS variant calling (STAR_OPOSSUM_PLATYPUS), and MAP-RSeq with RVBoost (MAPRSeq_RVBoost). MAP-RSeq use Tophat alignment and GATK unified genotyper. As Tophat is gapless alignment and RVBoost only works with SNVs, our comparison with this was limited to SNVs only. PanMutsRx obtained very similar sensitivities for both SNVs and Indels with more complexed Opossum pre-processing along with GATK haplotype caller (95% and 80% for SNVs and Indels, respectively). Opossum along with Platypus demonstrated the lowest sensitivities for SNVs and Indels, 86% and 75% respectively (Fig. 3). Although all combinations had very high specificity (> 99.98%), MAP-RSeq with RVBoost had the lowest number of false positive SNVs, which is not surprising as it applies more stringent filtering. The lower number of false positives for SNVs from Opossum preprocessing and PLATYPUS may explain its low sensitivity. Surprisingly, it also had the highest number of false positive Indels while its sensitivity was also the lowest (Fig. 4).

### Lung cancer adenocarcinoma RNA-seq dataset with known targetable/oncogenic mutations

The lung adenocarcinoma dataset SRA ERP001058 contains 41 tumors with known targetable or oncogenic mutations in 6 genes: EGFR, KRAS, NRAS, MET, BRAF and CTNNB1. The most notable are EGFR micro deletion at exon 19 (7 tumors) and single nucleotide substitution L858R at exon 21 (13 tumors) as they are targetable clinically. Among the 41 mutations, 39 were detected by the PanMutsRx pipeline with STAR aligner. Careful examination of the two tumors whose mutations were missed showed that both had very low mutation frequency (one with 1 and another with 3 mutated reads). As demonstrated in our previous evaluation, GSNAP is marginally more sensitive in indel detection compared to STAR, and this was corroborated using GSNAP for alignment, which indeed was able to detect both mutations as hypothesized (Table 1).

**Table 1:** Key known mutations of oncogenic genes detected by PanMutsRx

	Known	Detected	Sensitivity
BRAF V600E	1	1	1.00
CTNNB1 D32G	1	1	1.00
EGFR micro deletion	7	6 (7)	0.86 (1)^a^
EGFR SNV	14	13 (14)	0.93 (1)
KRAS SNV	14	14	1.00
MET SNV	1	1	1.00
NRAS SNV	3	3	1.00

^a^Numbers in parenthesis are from GSNAP alignment

### Gene fusions

**Fig. 5:**
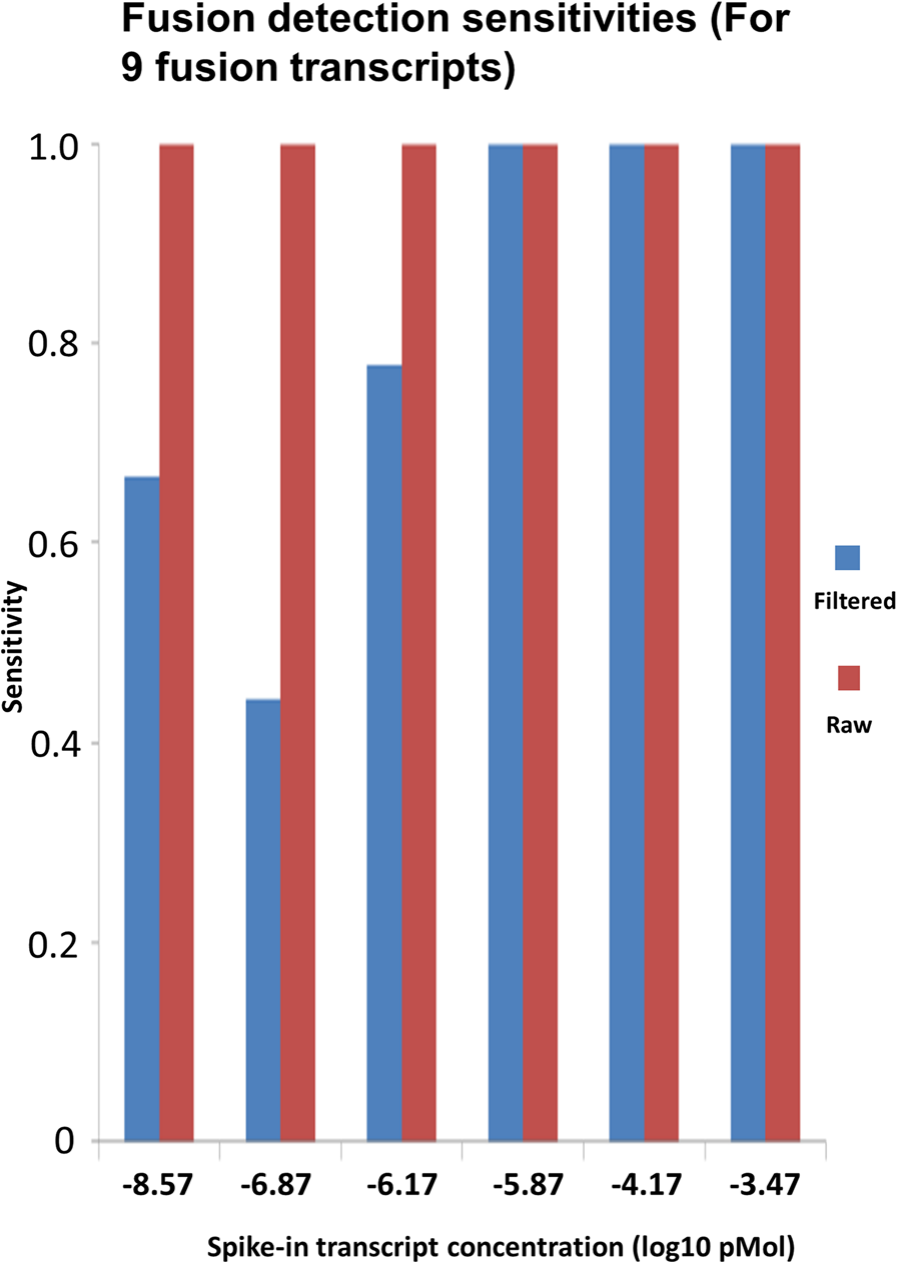
Fusion detection at different concentration of spike-ins known fusion transcripts. The fusions were called by STAR-Fusion. All expected fusions were detected from the raw output but fusions at low concentration were filtered. The filters applied include normalized split and spanning fragment reads > 0.1 FFPM, split reads with > = 25 bases at either side of a putative breakpoint

All 9 fusion transcripts were detected at each concentration from the initial detection output (raw result without strict filtering, Fig. 5). When filtering was applied, some fusions were filtered out for the library with a spike-in concentration below than or at − 6.17 (sensitivity ranging from 44 to 78%, Fig. 5). The trade-off certainly is between the sensitivity and specificity. For example, at the lowest concentration of − 8.57, 2 additional fusions were reported from the filtered result while the raw result had 16. In real practice, the filter stringency can be adjusted to balance the sensitivity and specificity.

### Gene expression quantification

**Fig. 6:**
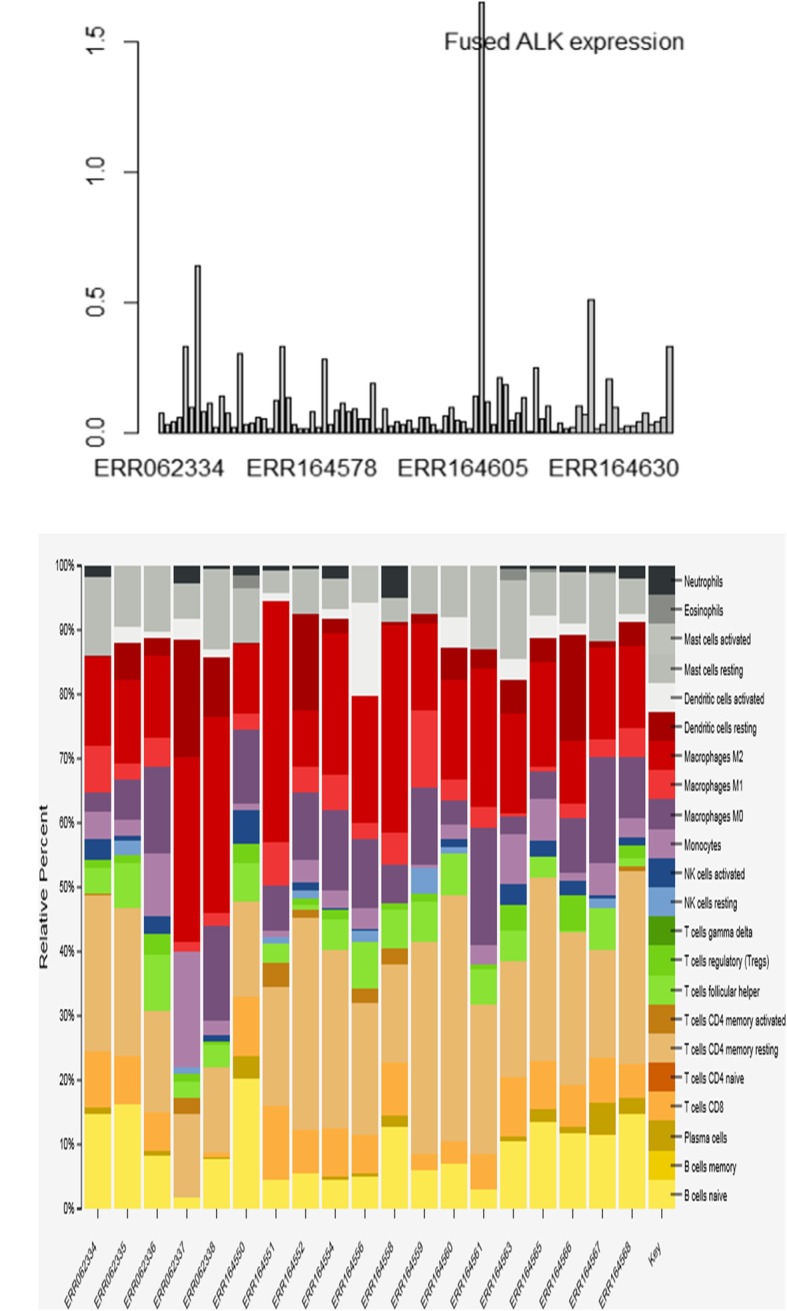
Application examples of gene expression data. **a** Outlier ALK expression as a result of EMLK4-ALK fusion in a tumor. **b** Estimation of immune cell relative proportions in the lung adenocarcinoma by Cibersort. Each stacked bar represents the percentages of immune cells in a tumor

Gene expression profiling is the most common analysis for RNA-seq and there is an array of approaches to further analyze the data. PanMutsRx generates two expression matrix files, raw digital read count and normalized expression by RPKM. The former can be used for differential expression by read count specific tools such as DESeq [29] or edgeR [30] and the latter can be used for linear model or comparing relative expression across genes. As an illustration, in the lung adenocarcinoma dataset, we also found a tumor with EML4-ALK fusion. Examining the expression of ALK across all tumors samples revealed that tumor had significantly higher expression of ALK (Fig. 6a), further validating the fusion led to the activation of ALK and was a potential candidate for targeted therapy by a protein kinase inhibitor such as Crizotinib. Another potential application from gene expression data is to estimate immune cell proportion in a tumor. Cybersort [31] is a tool using gene expression data to characterize cell composition of complex tissues. Based on a pre-built immune cell signature, it can be used to estimate the immune cell infiltration to a tumor that may provide useful information for an immune response status (Fig. 6b).

## Discussion

RNA-seq is one of the most commonly used sequencing applications as it measures the dynamics of genome transcription activities. Besides research, it also holds great promise for clinical diagnostics, prognostics and therapeutic applicability for various diseases, particularly cancers [32]. To put this into practice, various bioinformatics analyses challenges need to be overcome and to compile the types of information that can be reliably utilized for clinical applications from RNA-seq. Obviously, differential expression, alternative splicing, or allele specific expression are only unique to RNA-seq. RNA-seq is also an excellent platform for fusion transcript detection. The challenges are in detection of single nucleotide variants or small Indels from RNA-seq. Our previous evaluation shows that although SNVs can be reliably detected, indels are ignored by common RNA-seq tools, which calls for a need to develop a more sensitive pipeline [5]. PanMutsRx is developed to meet this specific and critical need.

PanMutsRx was designed with the goal of easy usage and detection of multiple types of mutations simultaneously. Our assessment showed its high sensitivity and specificity to SNVs and small Indels. Fusion transcripts can be easily detected and gene expression can be used along for cross validation of fusion transcript or other applications. In real practice of oncology, only very limited number of mutations has available drugs and capturing these mutations is of paramount priority. Our previous and current work suggests that although many unique mutations can be detected from either DNA-seq (like exome-seq) or RNA-seq, the important and actionable mutations are often conserved in RNA-seq. This suggests we can extract useful and relevant information to reduce the complexity of multi-genomic information from RNA-seq. We provide a post-processing script to extract SNVs, Indels, fusion transcripts, or expression for a list of genes users provide.

Available RNA-seq workflows mostly focus a particular function for example, gene expression, SNV or fusion transcript detection, which has its advantages of easy management. However, conducting analysis for each separately needs redundant work with significant effort for the RNA-seq data. PanMutsRx aimed to perform all clinical relevant tasks at once by selecting high performing tools for each application. RNA-seq alignment by different aligners makes much less difference for SNVs than for Indels and our selection of STAR and GSNAP as part of PanMutsRx was based on our comprehensive comparison among several tools [5]. Our current data further validated their good performance. For STAR alignment, it appears that PanMutsRx pre-processing generated very similar result as Opossum pre-processing. Results from GATK Haplotype caller were more sensitive than Platypus for both SNVs and Indels under the default settings. Parameter optimization may be needed to achieve better results. The slight gain from Opossum in some occasions may justify its adoption. As PanMutsRx is highly modular, a better tool can be integrated easily.

The missed calls in RNA-seq can be several reasons. We found majority of them were caused by insufficient alternative allele and although could be called but filtered out. These positions can be recovered by reducing filtering stringency but the trade-off would be increased false positives. Although Indel detection performs reasonably well, there is room for further improvement.

## Conclusion

We have developed a sensitive and comprehensive RNA-seq analytical pipeline which can capture multiple mutations simultaneously (single nucleotide, small insertion/deletion, chimeric transcripts or abnormal gene expression) and can be potentially used in clinical practice and precision medicine.

## Additional file

Additional file 1: **Table S1.** Parameter Settings used for alignment, data pre-processing and variant calling. **Table S2.** PanMutsRx Run time in each step of processing. (DOCX 18 kb)

## Abbreviations

FFPM: Fusion fragments per million total reads
FP: False positives
GIAB: Genome in a Bottle
Indels: Insertion/deletion
SNVs: Single nucleotide variants
TN: True negatives
