# Supplementary material for: Indel sensitive and comprehensive variant/mutation detection from RNA sequencing data for precision medicine
Source: BMC Med Genomics. 2018 Sep 14;11(Suppl 3):67. doi: 10.1186/s12920-018-0391-5 (PMC6157028; doi:10.1186/s12920-018-0391-5)
Supplement: Supplementary file 1 — Table S1. Parameter Settings used for alignment, data pre-processing and variant calling. Table S2. PanMutsRx Run time in each step of processing. (DOCX 18 kb) [file 12920_2018_391_MOESM1_ESM.docx]

## Supplementary Data

## Table S1. Parameter Settings for alignment, data pre-processing and variant calling

STAR+GATK Preprocess+Haplotype caller

STAR Alignment Options

STAR_OPTION_STEP2="--chimSegmentMin 12 --chimJunctionOverhangMin 12 --alignSJDBoverhangMin 10 --alignMatesGapMax 200000 --alignIntronMax 200000 --limitBAMsortRAM 31532137230 --outSAMstrandField intronMotif --outSAMtype BAM Unsorted --alignIntronMin 50 --sjdbGTFfile /data2/bsi/staff_analysis/m081429/Somatic_workflow_paper/ref_files/Homo_sapiens.GRCh37.75.mod.gtf --runThreadN 4 --sjdbOverhang 75 --twopassMode Basic --scoreDelOpen -1 --scoreDelBase -1 --scoreInsOpen -1 --scoreInsBase -1 "

GATK Proprocess Option

SplitNCigarReads (default options)

Realignment(default options)

IndelRealigner(default options)

BaseRecalibrator (below are the params)

GATK_BASE_RECALIBRATION_KNOWNSITES="-knownSites /data2/bsi/staff_analysis/m081429/Somatic_workflow_paper/ref_files/1000G_phase1.snps.high_confidence.hg19.sites.vcf.gz -knownSites /data2/bsi/staff_analysis/m081429/Somatic_workflow_paper/ref_files/Mills_and_1000G_gold_standard.indels.hg19.sites.vcf.gz -knownSites /data2/bsi/staff_analysis/m081429/Somatic_workflow_paper/ref_files/dbsnp_138.hg19.excluding_sites_after_129.vcf.gz"

GATK Caller options

GATK_HAPLOTYPE_CALLER_OPTION=" -stand_call_conf 20.0 -ERCIS 50 -pcrModel HOSTILE -stand_emit_conf 20.0 -mmq 20 -L /data2/bsi/staff_analysis/m081429/Somatic_workflow_paper/ref_files/coding.bed"

**GSNAP+GATK Preprocess+Haplotype caller**

GSNAP aligner option:

GSNAP_OPTION="-t 4 -A sam -D /data2/bsi/staff_analysis/m081429/Somatic_workflow_paper/ref_files/GSNAP -d GSNAP --use-splicing=/data2/bsi/staff_analysis/m081429/Somatic_workflow_paper/ref_files/GSNAP/GSNAP.maps/gencode.v19.splicesites.iit -N 1 --read-group-id=group1 --read-group-name=sample1 --read-group-library=lib1 --read-group-platform=illumina"

GATK Preprocess and Haplotype caller are the same as STAR alignment

Default settings were used for Opossum, Platypus and MAP-RSeq in comparisons.

## Table S2: PanMutsRx Run time in each step of processing

| Script | Runtime(secs) | min | Hours |
| --- | --- | --- | --- |
| Wrapper | 60 | 1 | 0.016667 |
| STAR Aligner | 16973 | 282.8833 | 4.714722 |
| GATK Preprocess | 45754 | 762.5667 | 12.70944 |
| GATK Variant calling | 10803 | 180.05 | 3.000833 |
| Fusion Calling | 427 | 7.116667 | 0.118611 |
| Feature count | 30 | 0.5 | 0.008333 |
| Cleanup | 600 | 10 | 0.166667 |
| Overall | 74647 | 1244.117 | 20.73528 |
